# TNF-α -308 A allele is associated with an increased risk of distant metastasis in rectal cancer patients from Southwestern China

**DOI:** 10.1371/journal.pone.0178218

**Published:** 2017-06-02

**Authors:** Zhen Li, Shu-an Li, Ya Sun, Yu Liu, Wen-liang Li, Li Yang, Yong Duan, Jingyu Li, Hao Guo, Tian-ning Zou, Yunlong Li, Kun-hua Wang

**Affiliations:** 1 Department of Gastrointestinal Surgery, Institute of Gastroenterology, The First Affiliated Hospital of Kunming Medical University, Kunming, Yunnan, China; 2 Kunming Digestive Disease Treatment Engineering Technology Center, Kunming, Yunnan, China; 3 Department of Gastroenterology, Institute of Gastroenterology, The First Affiliated Hospital of Kunming Medical University, Kunming, Yunnan, China; 4 Department of Breast Surgery, Yunnan Tumor Hospital, The Third Affiliated Hospital of Kunming Medical University, Kunming, Yunnan, China; 5 Department of Oncologic Surgery, The First Affiliated Hospital of Kunming Medical University, Kunming, Yunnan, China; 6 Department of Clinical Laboratory, Yunnan Tumor Hospital, The Third Affiliated Hospital of Kunming Medical University, Kunming, Yunnan, China; 7 Department of Clinical Laboratory, The First Affiliated Hospital of Kunming Medical University, Kunming, Yunnan, China; 8 Medical Faculty, Kunming University of Science and Technology, Kunming, Yunnan, China; 9 Department of Cardiology, The First Affiliated Hospital of Kunming Medical University, Kunming, Yunnan, China; 10 The First People’s Hospital of Yunnan Province (The Affiliated of Kunming University of and Technology), Kunming, Yunnan, China; University of South Alabama Mitchell Cancer Institute, UNITED STATES

## Abstract

Tumor necrosis factor-α (TNF-α), an important factor in systematic inflammation, is reportedly involved in several cancer types. The TNF-α -308 G>A (rs1800629) polymorphism in the promoter region influences TNF-α production. The association between TNF-α -308 G>A polymorphism and colorectal cancer (CRC) is not fully understood, especially the connections between TNF-α -308 G>A polymorphism and clinical features of CRC. In this study, TNF-α -308 G>A polymorphism was genotyped in 1140 individuals with or without CRC from Southwestern China. In case-control studies, we found no association between TNF-α -308 G>A polymorphism and CRC risk. Analysis of the correlations between TNF-α -308 G>A polymorphism and clinical features of CRC revealed that TNF-α -308 A allele was associated with higher body mass index (BMI) larger tumor size, and distant tumor metastasis in all CRC patients. Notably, rectal cancer (a subtype of CRC) patients with TNF-α -308 A allele had a very high risk of distant tumor metastasis [odds ratio (OR) = 4.481; 95% confidence interval (CI): 2.072–9.693; *P* = 0.00025]. The association between TNF-α -308 A allele and distant tumor metastasis remained even significant after adjusting all clinical characteristics (OR = 7.099; 95% CI: 2.482–20.301; *P* = 0.000256) in rectal cancer patients. Our results suggested that TNF-α -308 A allele was significantly associated with distant tumor metastasis in rectal cancer patients.

## Introduction

Colorectal cancer (CRC), one of the most prevalent malignant tumors, is the third most commonly diagnosed cancer and the fourth cause of cancer deaths worldwide [1]. The mortality and morbidity of CRC rapidly grows in the Chinese population. According to the latest Cancer Statistics of China [2], CRC is the fifth newly diagnosed cancer among men, and the fourth among women in China. CRC is considered to be a highly heterogeneous and polygenic disease[3, 4]. The tumorigenesis of CRC is still not completely clear and is thought to be a multistep event. Environmental factors and genetic susceptibility in different cohorts are accepted to play important roles in the tumorigenesis of CRC[5–7].

Distant metastasis adversely influences the survival of CRC patients [8] and significantly affects the treatment strategies of CRC. Inflammation and immune response play critical roles in CRC progression, including tumor metastasis and poor prognosis. The involvement of the immune system in CRC progression CRC is currently a research hotspot [9, 10]. Tumor necrosis factor-α (TNF-α) is an important pleiotropic cytokine produced by macrophages and is actively involved in immune regulations. TNF-α was thought to be associated with various malignant diseases, including cancer [11, 12]. The levels of elevated plasma TNF-α are commonly associated with poor prognosis [7, 13], such as tumor recurrence and positive lymph-node stage [14, 15]. Meanwhile, TNF-α can induce epithelial mesenchymal transformation and subsequently promotes the invasion and metastasis of colorectal cancer [16, 17]. Single nucleotide polymorphisms in TNF-α gene is reportedly associated with the cancer diagnosis, treatment outcome, and survival of cancer patients [18, 19]. For instance, TNF-α -308 polymorphism is significantly associated with the risk of gastric and hepatocellular carcinomas, triple-negative breast cancer, myeloma, and lymphoma [20–24], suggesting that TNF-α may be conducive to the early diagnosis of malignancies.

Some studies have focused on the association between TNF-α -308 polymorphism and CRC risk in different ethnicities and geographical regions worldwide [7, 24, 25]. However, results are inconsistent. Notably, research has been performed on associations between TNF-α -308 polymorphism and clinical features of CRC, especially in highly aggressive CRC with distant metastasis. In the present work, we conducted a case-control study to explore the role of TNF-α -308 polymorphism in CRC susceptibility, as well as the relationship between genotypes and clinicopathological characteristics.

## Patients and methods

### Ethics statement

Our study protocol was approved by the Institutional Review Board of Kunming Medical University. All study subjects provided written informed consent to participate in the study. The study conformed to the tenets of the Declaration of Helsinki. All methods applied were carried out in accordance with the approved guidelines.

### Study subjects

This case-control study included 570 patients with constitutive CRC and 570 cancer-free controls. All subjects were from southwestern China. Patients were recruited from January 2010 to December 2015 in the First Affiliated Hospital of Kunming Medical University & Yunnan Tumor Hospital and had been diagnosed with histologically confirmed CRC. We classified the BC subtypes as colon and rectal cancer. Controls were selected based on a physical examination in the same region during the same period as patient recruitment. The selection criteria included no history of cancer and frequency matching to cases by age and gender. At recruitment, demographic information and clinical characteristics of each participant were collected.

### Genotyping

DNA was extracted from leukocytes using a Wizard^®^ Genomic DNA Purification Kit (Promega Corporation, FL, USA) according to the manufacturer’s instructions. Genotyping of TNF-α -308 polymorphism (rs1800629) was performed using matrix-assisted laser desorption/ionization time-of-flight mass spectrometry. Primer sequences are available upon request.

### Statistical analysis

*Χ*^2^ tests was used to examine the deviation in genotype frequencies from Hardy–Weinberg equilibrium (HWE) in controls and was also used to analyze differences in demographic variables and genotype distributions for different clinical features of CRC. The association of TNF-α- 308 polymorphism with CRC incidence was calculated using adjusted odds ratios (AORs) and 95% confidence intervals (CIs) from multivariate logistic regression. The analysis was performed using SPSS 17.0 (SPSSInc., Chicago, IL, USA). *P* value less than 0.05 was considered statistically significant.

## Results

### Cases and controls

We had a total of 570 CRC cases, in which 250 were colon cancer, 316 were rectum cancer, and 4 were both with colon and rectal cancer (Table 1). The demographic characteristics of cases and controls are presented in Table 1.

**Table 1:** Baseline of colorectal cancer patients and control subjects.

Characteristics	Control	CRC^a^	*P*^*b*^
**Number**	570	570	
Age: years (mean±SDc)	58.5±14	59.1±11.6	0.46
**Gender**			
male (n%)	320(56%)	325(57%)	
famale (n%)	250(44%)	245(43%)	0.765
**Hypertension: n%**		137(24%)	
**Diabetes Mellitus: n%**		77(13.5%)	
**BMId≥25kg/m2: n%**		127(22.3%)	
**Tumor site: n%**			
Colon		250(43.9%)	
Rectum		316(55.4%)	
**Tumor size: n%**			
<5cm		376(66%)	
≥5cm		121(21.2)	
**TNMe stage: n%**			
I		73(12.8%)	
II		181(31.8%)	
III		156(27.6%)	
IV		137(24%)	

In this case-control study, the distributions of age and gender were consistently investigated among all cases and controls (P = 0.46 and 0.77, respectively). The genotype frequency of TNF-α-308 G and A alleles in the controls and cases concorded with HWE (P = 0.85 and 0.67, respectively). Considering that CRC is a complicated disease, many risk factors (such as age) are associated with the occurrence and progression of CRC [26, 27]. Therefore, we conducted stratified analysis on the data according to age, gender, tumor site, tumor size, tumor node metastasis (TNM) stage, presence of hypertension, diabetes mellitus, and levels of body Therefore, we conducted a stratified analysis on data according to age, gender, tumor site, tumor size, tumor-node-metastasis (TNM) stage, presence of hypertension, diabetes mellitus, and levels of body mass index (BMI) to determine whether the TNF-α-308 G>A polymorphism was associated with CRC incidence in specific subsets of the study population.

### TNF-α -308 G>A polymorphism and CRC

The frequency of TNF-α -308 A allele in CRC patients and relevant normal individuals was 0.070 and 0.067, respectively (Table 2). No significant difference in TNF-α -308 A and G alleles was observed between CRC cases and controls.

**Table 2:** Allele distribution of TNF-α-308 G and A allele in controls and patients with CRC.

Allele	Control	Case	OR95%	P
G	1061	1066	1	
A	79	72	0.907 (0.652–1.262)	0.613

Considering the few number of TNF-α -308 AA genotype, the dominant genetic model (GG/GA+AA) was used to analyze the genotype distribution of TNF-α -308 polymorphism. In the dominant genetic model, no significant (Table 3) difference between CRC cases and controls was found in TNF-α -308 polymorphism (OR = 0.884; 95% CI: 0.624–1.251; *P* = 0.535). TNF-α -308 polymorphism was not associated with the risk of colon cancer (OR = 0.895; 95% CI: 0.586–1.367; *P* = 0.661) or rectal cancer (OR = 1.386; 95% CI: 0.895–2.147; *P* = 0.165).

**Table 3:** Genotype distribution of TNF-α -308 G>A polymorphism in controls and patients with CRC.

Category	GG		GA+AA		OR (95% CI)	*P*
	n	(%)	n	(%)		
**Controls**	493	86.5	77	13.5	1	
**ALL CRC**	500	87.7	69	12.1	0.884(0.624–1.251)	0.535
**Tumor site**						
Colon	212	84.8	37	14.8	0.895(0.586–1.367)	0.661
Rectum	284	89.9	32	10.1	1.386(0.895–2.147)	0.165

### TNF-α -308 G>A polymorphism and clinical risk of CRC

To understand the association between TNF-α -308 G>A polymorphism and clinical features of CRC, logistic regression analysis was used to evaluate the correlations between TNF-α-308 G>A polymorphism and clinical characteristics (gender, age at diagnosis, BMI, tumor stage, tumor size, lymph-node metastasis, distant metastasis, history of polyp, and family history of cancer by CRC tumor-site classification), as shown in Table 4. In all CRC patients, the GA and AA genotypes were associated with higher BMI index (OR = 2.056; 95% CI: 1.197–3.530; *P* = 0.01) and larger tumor size (OR = 1.81; 95% CI: 1.029–3.183; *P* = 0.042). Notably, TNF-α -308 GA and AA genotypes showed strong association with distant tumor metastasis (OR = 2.911; 95% CI: 1.697–4.992; *P* = 0.00017). Furthermore, rectal cancer patients with TNF-α -308 A allele had a very high risk of distant tumor metastasis (OR = 4.481; 95% CI: 2.072–9.693; *P* = 0.00025), as shown in Table 4. After adjusting tumor stage, pathologic status, tumor size, and lymph-node metastasis status, the association became more significant (OR = 5.549; 95% CI: 2.437–12.632; *P* = 0.000045). The association between TNF-α -308 A allele and distant tumor metastasis remained even significant after adjusting all clinical characteristics (OR = 7.099; 95% CI: 2.482–20.301; *P* = 0.000256) in rectal cancer patients. By contrast, no association between TNF-α -308 GA and AA genotypes and distant metastasis was observed in colon-cancer patients (OR = 1.685; 95% CI: 0.592–4.796; *P* = 0.328). TNF-α -308 G>A was apparently not associated with other clinical features of CRC in our study.

**Table 4:** Clinicopathological features of CRC patients classified by tumor site and TNF-α -308 polymorphism. The total number of individuals may not be the same because of censored data.

Characteristics	ALL CRC(n = 570)				Colon cancer				Rectal Cancer			
	GG	GA+AA	OR(95%CI)	*P*	GG	GA+AA	OR(95%CI)	*P*	GG	GA+AA	OR(95%CI)	*P*
**Gender**												
famale(n = 245)	213	31			91	21			117	13		
male(n = 325)	287	38	0.91(0.548–1.51)	0.795	121	16	0.573 (0.283–1.16)	0.152	167	19	1.024(0.487–2.155)	1
**Age(years)**												
≤59(n = 257)	226	30			93	18			131	12		
>59(n = 313)	274	39	1.045(0.627–1.74)	0.798	119	19	0.825 (0.41–1.66)	0.596	153	20	1.427(0.672–3.029)	0.454
**BMI(kg/m2)**												
≤25(n = 443)	397	45			168	24			226	21		
>25(n = 127)	103	24	**2.056(1.197–3.530)**	**0.01**	44	13	2.068(0.975–4.388)	0.087	58	11	2.041(0.931–4.472)	0.111
**Tumour stage**												
I+II(n = 264)	226	37			95	22			129	15		
III+IV(n = 294)	262	30	0.699(0.419–1.169)	0.193	114	14	0.53(0.257–1.093)	0.104	146	16	0.942(0.448–1.982)	1
**Tumour size(cm)**												
≤5(n = 376)	334	41			130	17			203	24		
>5(n = 121)	99	22	**1.81(1.029–3.183)**	**0.042**	55	16	**2.225(1.049–4.719)**	**0.044**	42	6	1.208(0.465–3.138)	0.62
**Lymph node metastasis**												
NO(n = 296)	258	38			111	22			145	16		
YES(n = 256)	227	28	0.837(0.498–1.408)	0.514	97	13	0.676(0.323–1.414)	0.36	128	15	1.062(0.505–2.234)	1
**Distant metastasis**												
NO (n = 458)0	414	43			170	25			242	18		
YES(n = 112)1	86	26	**2.911(1.697–4.992)**	**0.00017**	42	12	1.943(0.903–4.182)	0.128	42	14	**4.481(2.072–9.693)**	**0.00025**
**Family history of cancer**												
NO(n = 525)	459	65			194	35			262	30		
YES(n = 45)	41	4	0.689(0.239–1.987)	0.637	18	2	0.616(0.137–2.773)	0.747	22	2	0.794(0.178–3.544)	1
**Smoking**												
NO(n = 443)	391	51			168	27			220	24		
YES(n = 127)	109	18	1.266(0.71–2.256)	0.441	44	10	1.414(0.637–3.141)	0.392	64	8	1.146(0.491–2.673)	0.824
**Alcohol**												
N0 (n = 490)	430	59			188	31			238	28		
YES(n = 80)	70	10	1.041(0.509–2.131)	1	24	6	1.516(0.574–4.007)	0.413	46	4	0.862(0.286–2.603)	1
**Hypertension**												
NO(n = 433)	381	51			162	29	0.894(0.384–2.08)	1	215	22		
YES(n = 137)	119	18	1.13(0.636–2.009)	0.764	50	8			69	10	1.416(0.639–3.137)	0.393
**Diabetes Mellitus**												
NO(n = 493)	434	58			181	33			249	25		
YES(n = 77)	66	11	1.247(0.623–2.498)	0.573	31	4	0.708(0.234–2.138)	0.797	35	7	1.992(0.802–4.947)	0.165
**Cokic Polyp**												
NO(n = 405)	360	45			153	27			205	18		
YES(n = 164)	140	24	1.371(0.805–2.336)	0.258	59	10	0.96(0.438–2.106)	1	79	14	2.018(0.958–4.252)	0.068

* The *P* value shown in Table did not make an adjustment by clinical characters.

### TNF-α -308 G>A polymorphism and physicochemical characteristics of CRC

TNF-α is an important cytokine related to immune regulation and inflammation. Therefore, the correlation of TNF-α -308 polymorphism with inflammatory, physiological, and biochemical indices was analyzed in CRC patients. The levels of neutrophil, leukocyte, cholesterol, triglyceride, albumin, high-density lipoprotein cholesterol (HDL-c), and low-density lipoprotein cholesterol (LDL-c) were used to access the relation with TNF-α -308 polymorphism in CRCs patients (Table 5). In all CRC patients, GA and AA genotypes were more frequent in patients with neutrophil percentage exceeding 75% than those with lower neutrophil percentage (OR = 4.764; 95% CI: 2.39–9.495; *P* = 0.000012). However, the association between TNF-α -308 G>A polymorphism and neutrophil percent disappeared in patients with rectal or colon cancer. TNF-α -308 G>A polymorphism was associated with the number of neutrophils, which may have resulted from inflammation caused by CRC. In addition to neutrophil percentage, other clinical feathers as listed in Table 5 were not associated with TNF-α -308 GA and AA genotypes.

**Table 5:** Physicochemical characteristic of CRC patients classified by tumor site and TNF-α -308 polymorphism. The total number of individuals may not be the same because of censored data.

Characteristics	ALL CRC(n = 570)				Colon cancer				Rectal Cancer			
	GG	GA+AA	OR(95%CI)	*P*	GG	GA+AA	OR(95%CI)	*P*	GG	GA+AA	OR(95%CI)	*P*
**Neutrophil (%)**												
≤75 (n = 439)	423	16			170	25			216	24		
>75 (n = 131)	111	20	**4.764(2.39–9.495)**	**0.000012**	42	12	1.943(0.903–4.182)	0.128	68	8	1.059(0.455–2.466)	0.831
**Cholesterol (mmol/L)**												
≤5.17 (n = 413)	359	54			144	28			213	26		
>5.17 (n = 156)	141	15	0.707(0.386–1.294)	0.314	68	9	0.681(0.304–1.522)	0.442	71	6	0.692(0.274–1.75)	0.52
**Triglyceride (mmol/L)**												
≤1.7 (n = 410)	360	50			150	27			206	23		
>1.7 (n = 159)	140	19	0.977(0.556–1.716)	1	62	10	0.896(0.409–1.962)	0.847	78	9	1.033(0.458–2.331)	1
**Albumin (g/L)**												
≥40 (n = 334)	290	44			124	24			164	20		
<40 (n = 235)	210	25	0.785(0.466–1.322)	0.434	88	13	0.763(0.369–1.581)	0.587	120	12	0.82(0.386–1.742)	0.707
**AST**^a^ **(U/L)**												
≤35(n = 506)	443	63			199	35			242	28		
>35(n = 63)	57	6	0.74(0.307–1.787)	0.551	13	2	0.875(0.189–4.045)	1	42	4	0.823(0.275–2.467)	1
**ALT**^b^ **(U/L)**												
≤40(n = 535)	470	65			203	36			265	29		
>40(n = 34)	30	4	0.964(0.329–2.825)	1	9	1	0.627(0.077–5.097)	1	19	3	1.443(0.403–5.172)	0.477
**HDL-c**^c^ **(mmol/L)**												
≥1.29(n = 161)	143	18			56	10			85	8		
<1.29(n = 408)	357	51	1.135(0.641–2.009)	0.674	156	27	0.969(0.441–2.13)	1	199	24	1.281(0.553–2.967)	0.684
**LDL-c**^d^ **(mmol/L)**												
≥2.7(n = 303)	279	34			125	22			151	12		
<2.7(n = 255)	221	35	1.3(0.785–2.151)	0.366	87	15	0.98(0.481–1.995)	1	133	20	1.892(0.891–4.017)	0.097

^a:^ glutamic oxalacetic transaminase;

^b:^ glutamic-pyruvic transaminase;

^c:^ high density lipoprotein cholesterol;

^d:^ low density lipoprotein cholesterol;

* The P value shown in Table did not make an adjustment by clinical characters.

## Discussion

TNF-α, an important inflammatory cytokine, is mainly produced by activated macrophages [28]. Recent studies have shown that TNF-α is actively involved in cellular apoptosis [29–31] and immune system[32–34]. TNF-α is also considered to be a very important connector between inflammation and cancer progression [35, 36]. These results suggested that TNF-α played a vital role in the etiology and progression of cancer. TNF-α -308 G>A polymorphism located in the promoter region reportedly influences TNF-α production [37]. Some studies have investigated the association between TNF-α -308 G>A polymorphism and CRC risk, but results are inconsistent [7]. Moreover, the association between TNF-α -308 G>A polymorphism and the clinical features of CRC is rarely studied. Therefore, from the point of view of population genetics, the role of TNF-α -308 G>A polymorphism in colorectal cancer requires further study.

To further understand the association between TNF-α -308 G>A polymorphism and CRC, 1140 individuals with or without CRC were enrolled in the present study. We found that TNF-α -308 G>A polymorphism showed no association with CRC risk in Han Chinese residing in Southwestern China, as shown in Tables 2 and 3. This result was generally consistent with meta-analysis results [32] indicating that TNF-α -308 G>A polymorphism does not confer susceptibility to CRC risk in Eastern populations. Notably, in Western populations, TNF-α -308 G>A polymorphism is thought to be a risk factor for CRC [32]. Therefore, our data further supported the notion that the genetic predisposition of TNF-α-308 G>A polymorphism to CRC was related to the substantial population heterogeneity.

The association between TNF-α -308 G>A polymorphism and the clinical features of CRC remains unclear. Recently, Li *et al* [24] suggested that the TNF-α-308 A allele is a risk factor of distant metastasis in patients with triple-negative breast cancer. This finding indicates that TNF-α -308 G>A polymorphism may be involved in cancer progression. In the present work, TNF-α -308 A allele was associated with higher BMI index, larger tumor size, and distant metastasis in CRC patients, as shown in Table 4. Our data showed that TNF-α -308 G>A polymorphism may be involved in cancer progression. Meanwhile, the correlation between TNF-α -308 A polymorphism and distant metastasis remained in rectal cancer patients (Table 4), indicating that TNF-α -308 G>A polymorphism may be an important risk factor affecting rectal-cancer patients. The potential role of TNF-α 308 A allele in CRC metastasis requires further verification through animal experiments.

TNF-α -308 G>A polymorphism was associated with the number of neutrophils in all CRC patients (Table 5), indicating that inflammation in CRC patients may be related to TNF-α -308 G>A polymorphism. However, the association between TNF-α -308 G>A polymorphism and the number of neutrophils cannot be further detected in CRC subtypes (rectal and colon cancers), as shown in Table 5. The association between TNF-α-308 G>A polymorphism and neutrophils may be unrelated to neoplasm location (colon or rectum), although further confirmation is necessary.

There are some limitations in this study. First, the sample size is modest in the present study. It could not fully detect the susceptibility of TNF-α -308 G>A polymorphism in each subtypes of CRC. Second, the results of the present study did not make a replicated study in another cohort of CRC patients. It should be further verified. Finally, the functional study was not performed to determine the role of TNF-α -308 A allele in distant metastasis of CRC subtypes.

Generally, in Han Chinese of southwestern China, TNF-α -308 G>A polymorphism may not predispose to CRC risk, but it may significantly increase risk of distant metastasis in rectal cancer patients.

## Supporting information

S1 File Genotype and clinical information for CRC patients in the present study. (ZIP)
